# 1304. Mission-based Academic Tweeting: Analysis of Content Engagement over One Year

**DOI:** 10.1093/ofid/ofac492.1135

**Published:** 2022-12-15

**Authors:** Teena Xu, Emmanuel Guajardo

**Affiliations:** Baylor College of Medicine, Houston, Texas; New Orleans VA Medical Center, New Orleans, Louisiana

## Abstract

**Background:**

The Baylor College of Medicine Infectious Diseases Fellowship program (BCM ID) launched a new academic Twitter account during the COVID-19 pandemic with the mission to promote the achievements of fellows and faculty (promotion-based tweets, or PBT) and disseminate original educational material (education-based tweets, or EBT) during a fellowship recruitment season that became virtual due to the pandemic. Account content was developed by both ID fellows and faculty, with the goal of increasing social media engagement with the fellowship program. Currently, the average Twitter engagement rate per tweet is 0.037% across all industries and 0.7% for higher education accounts. We looked at the engagement rate of mission-based tweets during the inaugural year of the BCM ID Fellowship twitter account.

**Methods:**

We conducted a retrospective review of tweets published on the BCM ID Fellowship account during the first year of operations (8/1/20-7/31/21). We reviewed 64 mission-based tweets for impressions (number of times a tweet is shown to users), engagement (number of times users interacted with a tweet), and reach (number of followers). We calculated engagement rate (engagement-to-impression ratio, or E:I) for each tweet and compared the engagement rates between EBT and PBT. We also examined the trend of followers over time. Data were collected in October 2021.

**Results:**

EBT comprised 41% of total tweets, and PBT comprised the remaining 59%. EBT averaged 3,662 impressions and 458 engagements, for an E:I of 9.6%. PBT averaged 2,449 impressions and 130 engagements, for an E:I of 5.3% (p = 0.007). Despite a decrease in total posts per month over this time period, follower count continued to increase, and monthly engagement rate per tweet remained above 1%.

**Figure:**
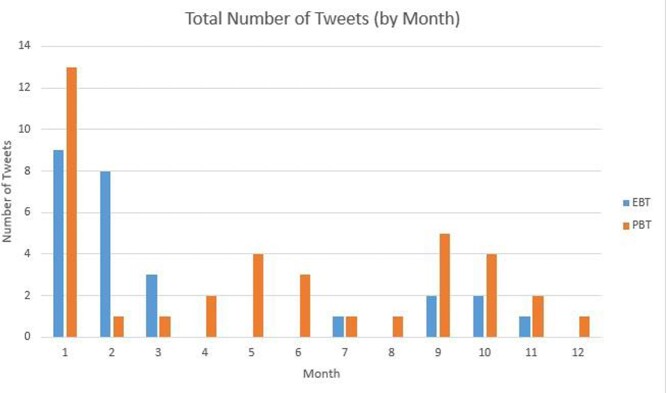

**Figure:**
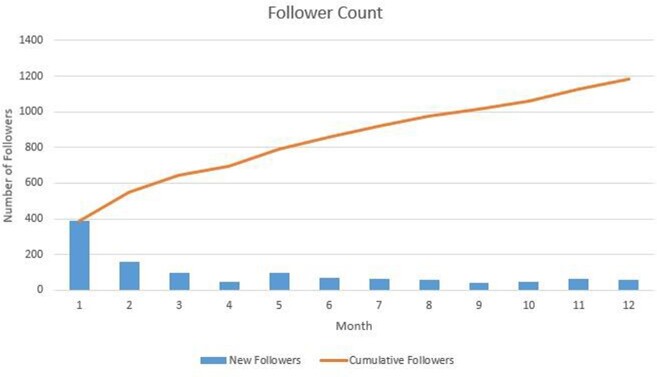

**Figure:**
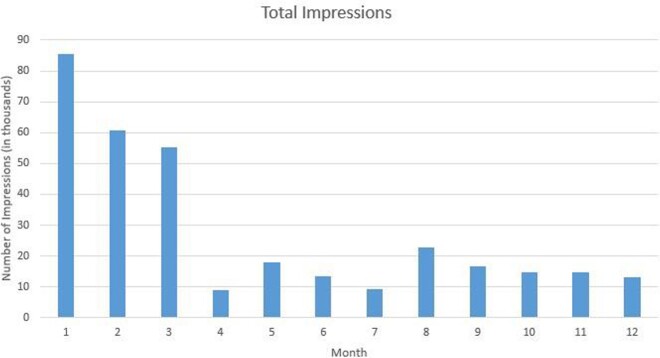

**Conclusion:**

Our experience suggests that user engagement is higher for EBT. Programs planning to launch a new academic Twitter account should consider focusing initial content on education to maximize overall engagement and reach. One limitation of this study is that frequency of tweets also impacts engagement, which may confound data from later in the inaugural year, when tweeting from the account became less frequent.

**Figure:**
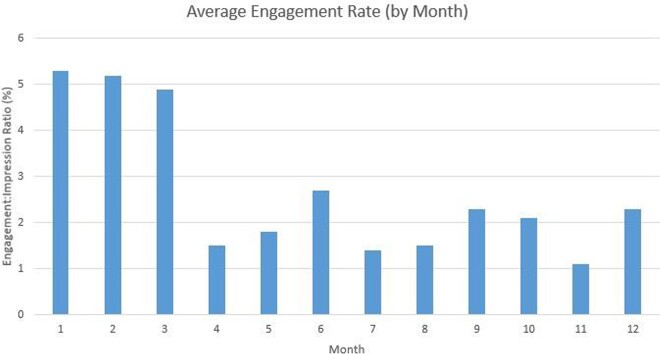

**Disclosures:**

**All Authors**: No reported disclosures.

